# Clinical outcomes and prognostic factors of robotic assisted rectal cancer resection alone versus robotic rectal cancer resection with natural orifice extraction: a matched analysis

**DOI:** 10.1038/s41598-020-69830-1

**Published:** 2020-07-30

**Authors:** Dongning Liu, Rui Luo, Zhikai Wan, Weiquan Zhu, Penghui He, Shanping Ye, Cheng Tang, Xiong Lei, Taiyuan Li

**Affiliations:** 1 Department of General Surgery, The First Affiliated Hospital of Nanchang University, No. 17, YongWaiZheng Street, Nanchang, 330000 China; 2 Medical College of Nanchang University, Nanchang, 330000 China

**Keywords:** Rectal cancer, Cancer therapy

## Abstract

Robotic rectal cancer resection with natural orifice extraction is a recently developed minimally invasive surgery used in the treatment of patients with rectal cancer. However, its safety and feasibility remain undiscussed and controversial. This study reported the clinical outcomes and prognostic factors pertaining to traditional robotic assisted rectal cancer resection alone against that of robotic rectal cancer resection with natural orifice extraction to provide a discussion on this issue. 49 patients who underwent robotic rectal cancer resection with natural orifice extraction and 49 matched patients who underwent conventional robotic assisted rectal cancer resection were systematically analyzed in this study. Regarding the baseline characteristics, after matching, no significant differences were observed between the natural orifice specimen extraction (NOSE) group and the robotic assisted rectal cancer resection (RARC) group. Patients in the NOSE group had a reduced visual analog scale (*p* < 0.001), passed flatus more quickly (*p* = 0.002) and suffered less surgical stress than those in the RARC group. Moreover, 4 complications were observed in the NOSE group and 7 complications in the RARC group with no significant difference (*p* = 0.337) in terms of complications. The two groups had a similar survival outcomes, where the 3-year overall survival (*p* = 0.738) and 3-year progression-free survival (*p* = 0.986) were all comparable between the two groups. Histological differentiation and T stage could be regarded as independent prognostic factors for 3-year overall survival and 3-year progression-free survival. Robotic rectal cancer resection with natural orifice extraction is a safe and feasible minimally invasive surgery for patients suffering from rectal cancer as it encompasses considerable several advantages. Histological differentiation and T stage may serve as independent prognostic factors for 3-year overall survival and 3-year progression-free survival.

## Introduction

Colorectal cancer is the fifth most commonly diagnosed cancer and the fifth leading cause of cancer death with nearly 3,763,000 patients diagnosed with colorectal cancer in China in 2015. This number is estimated to increase annually according to the latest cancer statistics in China^1^. Surgical treatment modalities are a major role in the treatment of rectal cancer and is the cornerstone of curative treatment^2^. With total mesorectal excision serving as the standard oncological approach, surgical methods for rectal cancer are gradually becoming enriched and more minimally invasive, and they are roughly divided into laparotomy, laparoscopic surgery, transanal endoscopic microsurgery and robotic surgery^3^. By being able to filter physiological tremors, robotic rectal cancer surgery can distinctly reduce vascular nerve injury^4^. However, the abdominal incision to extract the specimen has been annoying the surgeon and patients for postoperative pain, surgical wound infection, incisional hernia, postoperative scarring and so on in the era of minimally invasive. However, the so-called natural orifice specimen extraction concept applied in the robotic assisted rectal resection can give surgeons and patients a satisfactory result theoretically^4^. This approach extracts the specimen from the anus, hence the abdominal incision in the robotic assisted rectal resection can be eliminated^5,6^. Since Franklin Jr et al. probably first proposed that for small specimen in sigmoid resection the transanal route is desirable^7^, the natural orifice extraction specimen concept has gained tremendous attention and a series of cases have been reported thereafter. However, most such studies are limited to case reports, animal experiments, and are small in number^8–12^. Notwithstanding, few studies have compared conventional robotic assisted rectal cancer resection and robotic rectal cancer resection with natural orifice extraction in terms of neither short-term outcomes nor long-term outcomes.

Since robotic gastrointestinal surgeries have been carried out at our institution since 2014 and the first natural orifice specimen extraction surgery via laparoscopy was performed in 2008, this study was conducted to compare the short-term and long-term outcomes between conventional robotic assisted rectal cancer resection and robotic rectal cancer resection with natural orifice extraction in order to assess the effectiveness and safety of robotic rectal cancer resection with natural orifice extraction.

## Material and methods

### Patients

**Figure 1:**
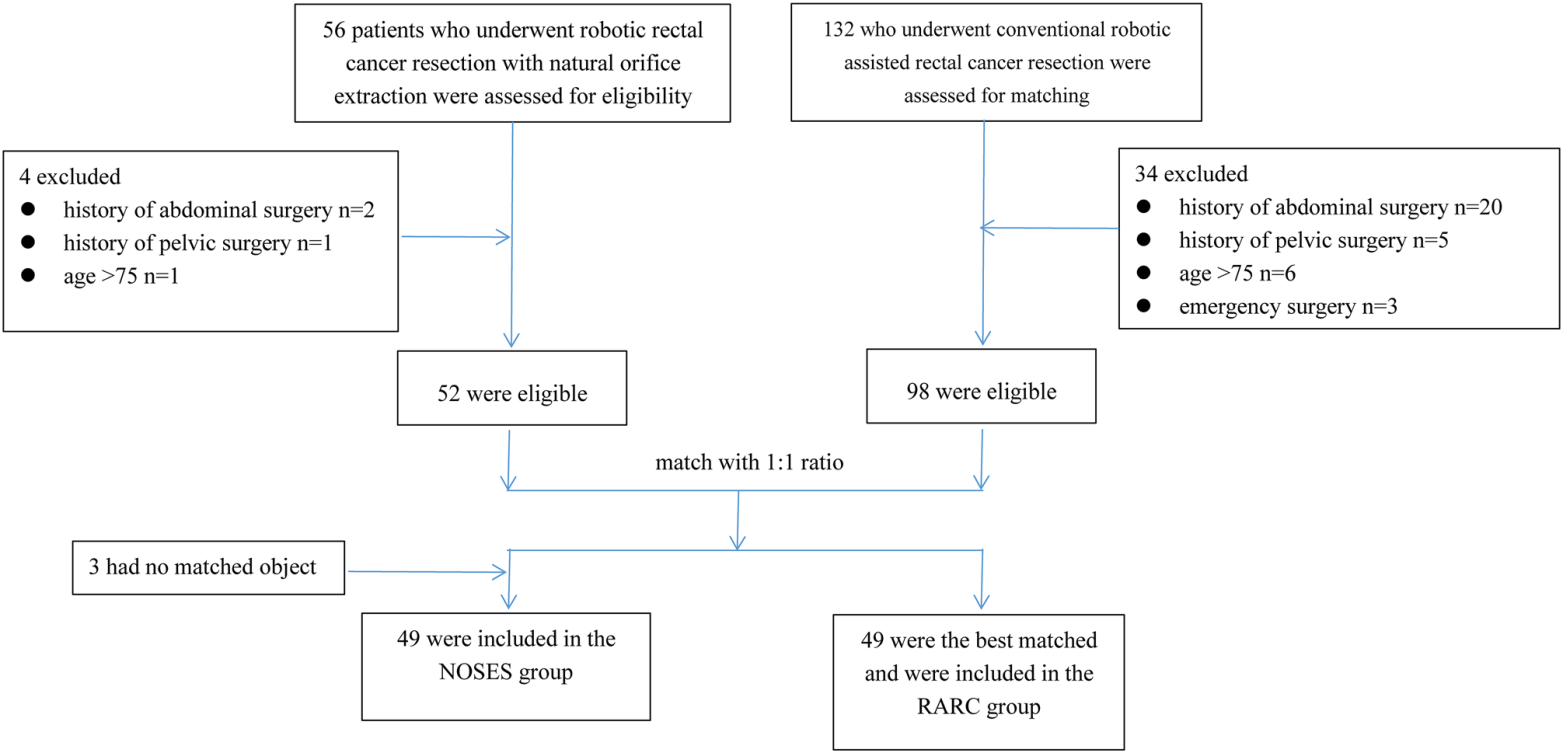
Patient flowchart.

From January 2015 to November 2016, 56 patients underwent robotic rectal cancer resection with natural orifice extraction while 132 patients underwent conventional robotic assisted rectal cancer resection. Patients were included and excluded according to the following indications. The inclusion criteria were: (1) T stage 1–3; (2) aged between 18 and 75; (3) tumor margin being at least 4 cm from the anus; (4) body mass index ≤ 30 kg/m^2^; (5) no distant metastases; and (6) tumor size ≤ 5 cm. The exclusion criteria were: (1) emergency surgery for intestinal obstruction or massive bleeding; and (2) history of abdominal or pelvic surgery. Finally, 52 patients who underwent robotic rectal cancer resection with natural orifice extraction and 98 who underwent conventional robotic assisted rectal cancer resection were enrolled. Accordingly, 3 patients among the 52 patients were not matched out of the 98 patients. Hence, 49 patients were included in the NOSE group while 49 patients were included in the RARC group. The matching confounding factors included age (± 2 years), gender, date of surgery (± 2 months), body mass index (± 1 kg/m^2^), T stage, and tumor size (± 1 cm). Moreover, the included patients had no emergency surgery for intestinal obstruction or massive bleeding, and they did not have a history of abdominal or pelvic surgery with no missing information. For certain patients in the NOSE group, if two or more were matched according to the above-mentioned criteria, the patient who had the most appropriate tumor size to the corresponding one was selected. The corresponding flowchart is presented in Fig. 1. The matching process was accomplished by a trial coordinator independent to the study. Patients with T3 and N+ were recommended to receive preoperative neoadjuvant chemoradiotherapy. Limited by the local economic level local socio-economic factors, few patients received preoperative neoadjuvant chemoradiotherapy. All patients received preoperative assessment so as to determine surgical resection indications such as physical examinations, serum biochemical examinations, colonoscopic biopsies and radiographical examinations like abdominal computed tomography and pelvic magnetic resonance imaging, if applicable. T and N stages were classified using the 8th edition of the UICC/AJCC TNM staging system for colorectal cancer, which were confirmed according to the examinations. All these procedures in the study were complied with the Declaration of Helsinki, and the study was approved by the ethics committee of the First Affiliated Hospital of Nanchang University. Hence, Informed consent from patients for this study was waived. Patient-controlled analgesia (PCA) was applied for all the patients. If the PCA was inadequate in controlling the pain, intravenous analgesia was given (flurbiprofen), which was infused intravenously 100 mg per day. Patients who underwent conventional robotic assisted rectal cancer resection were assigned to the RARC group, while patients who underwent robotic rectal cancer resection with natural orifice extraction were assigned to the NOSE group.

### Surgeon background

All operations were performed by an experienced surgeon with more than ten years of experience in laparoscopic gastrointestinal surgery, having done over 1,500 robotic gastrointestinal operations. All operations were performed in accordance to the total mesorectal excision principle.

### Statistical analysis

Data were expressed as means with standard deviation or number. Continuous data was compared using the Mann–Whitney test, and categorical data was compared using the Fisher’ exact or χ^2^ tests. As appropriate, the Kaplan–Meier method was used to compare overall survival and progression-free survival, while Log-Rank test was used to compare the differences between the NOSE group and RARC group for overall survival and progression-free survival. Data analysis was done via SPSS 22.0 (SPSS, Chicago, USA). The survival curves were drawn using R studio (version: 1.2.5033) with the packages survminer, ggplot2, ggpubr, magrittr and survival. *p* < 0.05 was considered to be statistically significant.

### Endpoints

The primary endpoints for this current study were overall survival, defined as the time from the date of surgery to death, and progression-free survival which is defined as the time from the date of surgery to documented any death or disease recurrence including locoregional recurrence and distant metastasis. Patients who were lost to follow-up and were still alive at the last follow-up would be documented as censored data in terms of overall survival and who were lost to follow-up and still alive without recurrence at the last follow-up. The secondary endpoints for this current study is short-term efficacy and safety. The parameters to assess short-term efficacy and safety include operation time, estimated intraoperative blood loss, postoperative visual analog scale (VAS) on day 1, postoperative use of analgesics, time to pass flatus, postoperative hospital stay, hospitalization costs, postoperative complications, postoperative white blood cell count, postoperative procalcitonin, and postoperative C-reactive protein. Postoperative white blood cell count level, postoperative procalcitonin level and postoperative C-reactive protein level were tested on postoperative day 1, 3 and 5 to assess the surgical stress response. Postoperative VAS scale was tested on postoperative day 1.

### Surgical technique

Patients adopted the lithotomy position with their right thigh slightly flat and were under general anesthesia. All cases adopted the four-trocar position with a pneumoperitoneum of 15 mmHg. Meanwhile, a 12 mm camera trocar was placed 3 cm above the umbilicus and 3 cm at the right side. The right McBurney point was disposed into an 8 mm trocar as the first robotic manipulator connected to an ultrasound knife. The anti- McBurney point was disposed into an 8 mm trocar as the third manipulator connected to a bipolar electric coagulation gripper. At 8 cm above the anti- McBurney point, a 12 mm trocar was placed as the assistant hole. First, the inferior mesenteric arteriovenous roots were dissociated and severed. Then, the mesentery was freed of sufficient length according to the location of the tumor while attention was given to the protection of reproductive vessels and ureters. Afterward, nake bowel beneath the tumor about 3–5 cm and nake about 2 cm on the pre-resection line on the sigmoid. An assistant then inserted a sterile protective plastic sheath into the abdominal cavity through the assistant hole. After another assistant fully expanded the anus and disinfected and washed the rectum enteric cavity fully with iodine saline, the distal rectum was transected through the naked bowel beneath the tumor via ultrasound knife, and the sterile plastic sheath was pulled out using oval forceps inside the anus. An anvil head of a circular stapler was placed in the abdomen through the distal rectal stump and inserted in into the proximal sigmoid wall through a 2 cm longitudinal incision beneath the pre-resection line, after which the sigmoid stump was adequately disinfected with iodine saline. Next, the sigmoid was transected through the pre-resection line using a linear stapler. Here, the tumor specimen was completely dissociated in the abdominal cavity. Specimen extraction was different between the NOSE group and RARC group. In the NOSE group, the specimen was extracted through the anus using oval forceps through the inserted sterile plastic sheath. In the RARC group, the specimen was extracted through a 5 cm incision in the hypogastrium. The distal rectal opening was then closed using a linear stapler, and end-to-end anastomosis was performed using a circular stapler while the “danger triangle” was sutured. Finally, the abdominal cavity was thoroughly cleaned and washed to prevent tumor cells from remaining. The anastomosis was ensured to be unobstructed and had no leakage. At this point, the digestive tract reconstruction was completed, and a drainage tube was placed in the pelvic cavity and extracted from the right lower abdomen, after which the abdomen was closed layer-by-layer.

### Follow-up

In order to eliminate possible micrometastases and improve patient survival, patients received postoperative chemotherapy postoperatively. Patients diagnosed with T1-2N0M0 received no chemotherapy, while patients diagnosed with T3N0M0 received a 5 fluorouracil / calcium folinate/oxaliplatin regimen. Additionally, patients diagnosed with T1-3N1-2M0 received the FOLFOX regimen. Patients visited the outpatient department every three months in the first two years and every six months thereafter. The patients received physical examinations and tumor biomarkers investigations at every outpatient visit along with chest X-ray examinations and abdominal and pelvic CT scans at least once a year. At the same time, the patients were followed up via WeChat or telephone on a regular basis. The last follow-up day was November 30, 2019.

## Results

### Baseline characteristics

All surgeries were performed successfully. The baseline characteristics are summarized in Table 1. Prior to matching, the frequencies of T stage in the NOSE group were 10, 12 and 30 for I, II, and III, while that in the RARC group were 12, 45 and 41. The composition of T stage in the two groups had a significant difference with a *p* value of 0.022. After matching, no significant differences were observed in the two groups. In terms of postoperative pathological results, likewise specimen lengths were resected in both groups (15.9 ± 2.3 cm vs. 15.2 ± 2.4 cm *p* = 0.091). The proximal margin in the NOSE group was found to be marginally longer than that in the RARC group (8.3 ± 2.5 cm vs. 4.4 ± 1.2 cm *p* = 0.050), which led to the marginally shorter distal margin in the NOSE group (3.9 ± 1.0 cm vs. 4.4 ± 1.2 *p* = 0.058). The CRM positive rates were about the same in both groups (2.0% vs. 4.1%, *p* = 0.558). Histological differentiation showed no significant differences in both groups (*p* = 0.453), and the number of lymph nodes harvested was similar in both groups (20.6 ± 6.5 vs. 19.7 ± 6.5 *p* = 0.647). Although it is recommended to receive neoadjuvant chemoradiotherapy in patients having T3 and N+ rectal cancer, only few patients received it due to financial reasons. Preoperative serum CA19-9 and CEA were evaluated in both groups and compared to normal levels, showing no differences in both groups, respectively. These results demonstrate that the following short-term results and long-term results are comparable.

**Table 1:** Baseline characteristics.

Baseline characteristics	Before matching			After matching		
	NOSE group (n = 52)	RARC group (n = 98)	*p*	NOSE group (n = 49)	RARC group (n = 49)	*p*
Age (year)	57.2 ± 10.2	56.5 ± 9.2	0.458	57.1 ± 10.4	55.4 ± 9.3	0.522
Gender			0.721			1.000
Male	26	52		25	25	
Female	26	46		24	24	
BMI (cm/kg^2^)	23.2 ± 2.7	24.0 ± 2.5	0.856	23.1 ± 2.6	23.0 ± 2.4	0.904
ASA score			0.106			0.584
I	2	14		1	2	
II	42	66		40	42	
III	8	18		8	5	
Tumor size (cm)	3.9 ± 0.9	3.6 ± 0.8	0.393	3.8 ± 0.9	3.6 ± 0.8	0.393
Distance from the tumor margin to the anus (cm)	9.6 ± 2.4	8.6 ± 2.7	0.055	9.6 ± 2.3	8.6 ± 2.7	0.056
Specimen length (cm)	15.3 ± 2.2	15.1 ± 2.1	0.103	15.9 ± 2.3	15.2 ± 2.4	0.091
Proximal margin (cm)	8.2 ± 2.3	4.5 ± 1.3	0.085	8.3 ± 2.5	4.4 ± 1.2	0.050
Distal margin (cm)	3.8 ± 1.1	4.3 ± 1.3	0.069	3.9 ± 1.0	4.4 ± 1.2	0.058
CRM positive	1	3	0.680	1(2.0%)	2(4.1%)	0.558
Histological differentiation			0.564			0.453
Well	9	11		8	6	
Moderate	39	80		39	38	
Poor	4	7		2	5	
Number of lymph nodes harvested (n)	20.5 ± 6.5	19.9 ± 6.4	0.685	20.6 ± 6.5	19.7 ± 6.5	0.647
Preoperative serum CA19-9 (U/ml)	23.6 ± 13.5	21.4 ± 12.0	0.447	23.7 ± 13.5	21.4 ± 12.0	0.447
Preoperative serum CEA (ng/ml)	5.4 ± 3.2	6.4 ± 4.1	0.283	5.4 ± 3.1	6.4 ± 4.1	0.291
T stage			**0.022**			1.000
I	10	12		9	9	
II	12	45		10	10	
III	30	41		30	30	
N stage			0.144			0.811
I	19	21		18	15	
II	26	56		24	26	
III	7	19		7	8	
Preoperative chemoradiotherapy	8	17	0.836	7	9	0.585

*p* < 0.05 value is indicated in bold.

*BMI* body mass index, *ASA score* American society of anesthesiologists score.

### Short-term efficacy and safety outcomes

Table 2 summarizes the short-term outcomes between the two groups. The operation time was fundamentally identical in both groups (NOSE group 164.7 ± 29.1 min vs. RARC group 154.7 ± 27.2, *p* = 0.124). In all 98 cases, no patient required conversion to open surgery. Moreover, the NOSE group and RARC group had similar estimated intraoperative blood loss (66.2 ± 22.8 ml vs. 71.9 ± 17.8 ml *p* = 0.214), indicating that vascular damage was similar in both groups. In terms of postoperative recovery indicators, patients in the NOSE group suffered significantly less pain than those in the RARC group (3.9 ± 1.1 vs. 5.5 ± 1.1 *p* < 0.001) while fewer patients required analgesia (5/49 vs. 15/49, *p* = 0.012). Additionally, time to pass flatus was shorter in the NOSE group than in the RARC group (2.8 ± 0.8 d vs. 3.6 ± 0.8 d *p* = 0.002). As for financial burden, patients in both groups paid similar hospital costs (9,352.2 ± 799.5$ vs. 9,423.7 ± 465.8 *p* = 0.529) and stayed in the hospital in the same conditions (7.5 ± 1.7 vs. 7.3 ± 1.3 *p* = 0.533). In regard to surgical stress response, postoperative white blood cell count and postoperative procalcitonin showed no significant differences on postoperative days 1, 3 or 5. Concerning postoperative complications, 4 complications were observed in the NOSE group while 7 complications were noted in the RARC group (*p* = 0.337). Accordingly, 1 anastomotic leakage, 2 intra-abdominal abscess and 1 ileus in the NOSE group and 2 anastomotic leakages, 1 bleeding of anterior sacral, 2 wound infections, 1 intra-abdominal abscess and 1 ileus in the RARC group were observed overall. However, postoperative C-reactive protein on postoperative days 1 and 3 was significantly reduced in the NOSE group than in the RARC group and showed no significant differences in both groups on postoperative day 5. The values of the mentioned parameters as well as the *p*-value are summarized in Table 2.

**Table 2:** Short-term outcomes.

Parameters	NOSE group	RARC group	*p*
Operation time (min)	164.7 ± 29.1	154.7 ± 27.2	0.124
Estimated intraoperative blood loss (ml)	66.2 ± 22.8	71.9 ± 17.8	0.214
Postoperative VAS scale on day 1	3.9 ± 1.1	5.5 ± 1.1	**< 0.001**
Additional use of analgesics (n)	5	15	**0.012**
Time to pass flatus (d)	2.8 ± 0.8	3.6 ± 0.8	**0.002**
Postoperative hospital stay (d)	7.5 ± 1.7	7.3 ± 1.3	0.553
Hospitalization costs ($)	9,352.2 ± 799.5	9,423.7 ± 465.8	0.529
Postoperative complications	4	7	0.337
Anastomotic leakage	1	2	
Bleeding of anterior sacral	0	1	
Wound infection	0	2	
Intra-abdominal abscess	2	1	
Ileus	1	1	
Postoperative white blood cell count (/l)			
Day 1	12.7 ± 3.3	13.3 ± 2.4	0.422
Day 3	10.4 ± 2.1	9.7 ± 2.1	0.091
Day 5	7.4 ± 1.5	7.1 ± 1.5	0.436
Postoperative procalcitonin (ng/ml)			
Day 1	6.3 ± 3.1	8.0 ± 4.4	0.356
Day 3	6.0 ± 2.8	6.7 ± 3.1	0.486
Day 5	2.7 ± 1.8	3.4 ± 1.3	0.358
Postoperative C-reactive protein (mg/l)			
Day 1	64.2 ± 17.8	72.8 ± 18.0	**0.020**
Day 3	72.9 ± 10.9	82.5 ± 10.4	**< 0.001**
Day 5	47.4 ± 10.5	49.0 ± 8.9	0.268

*p* < 0.05 values are indicated in bold.

*CRM* circumferential resection margin.

### Survival analysis

**Figure 2:**
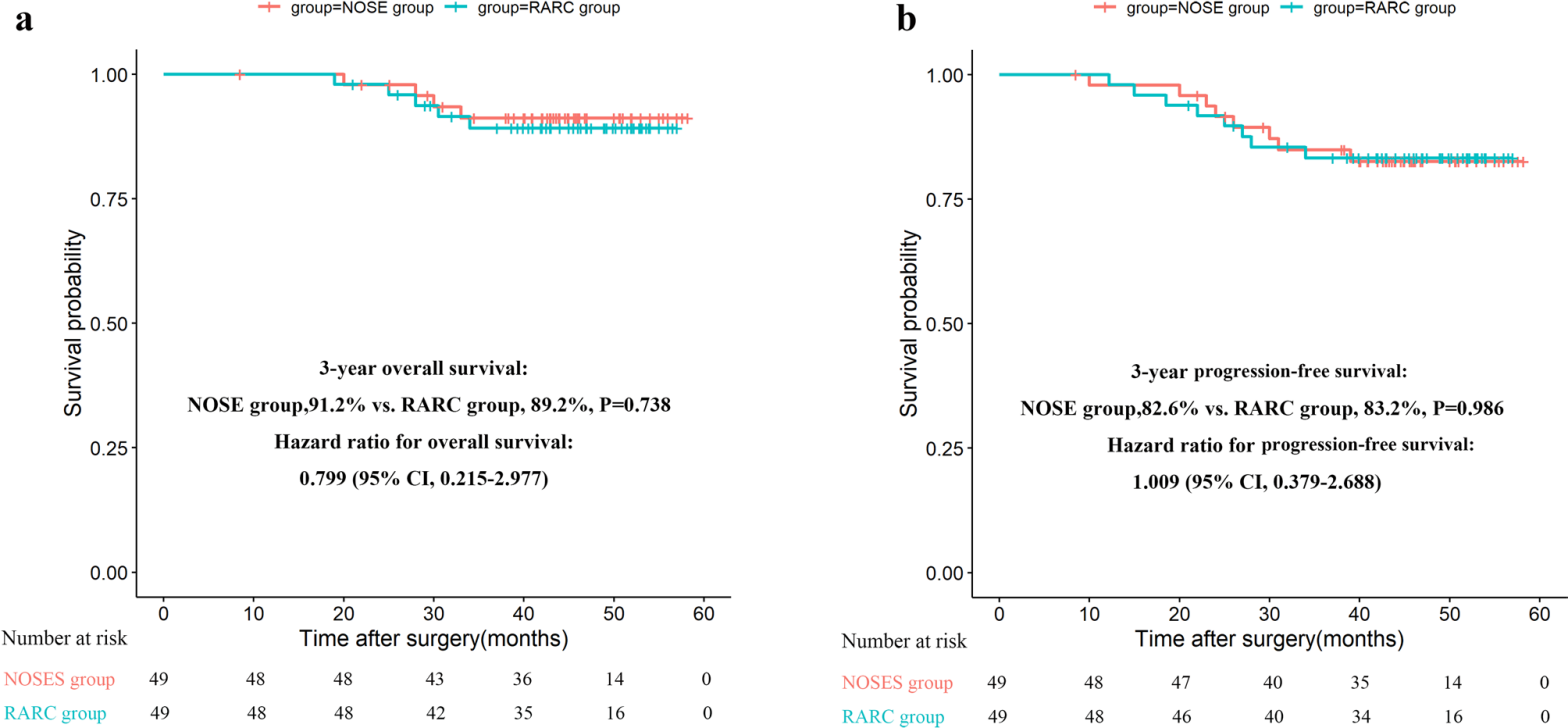
(**a**) Overall survival. (**b**) Progression-free survival.

The Kaplan–Meier curves are shown in Fig. 2a,b. At the last follow-up on November 30, 2019, the median follow-up time was 42.7 months (range from 8 to 58.2) in the NOSE group and 43.7 months (range from 19 to 57) in the RARC group. A total of 39 among the 49 patients (80%) were followed up for more than 36 months in both groups. The 3-year overall survival in the NOSE group was 91.2% while that of the RARC group was 89.2%. Additionally, the Log-rank test and Cox proportional hazard model showed no significant differences between the two groups (*p* = 0.738, HR = 799, 95% CI 0.215–2.977), and no significant differences were observed in 3-year progression-free survival (82.6% vs. 83.2%, *p* = 0.986, HR = 1.009, 95% CI 0.379–2.688). In the NOSE group, two patients developed local recurrence while 6 patients developed distant recurrence (4 in the liver and 4 in the lung). In the RARC group, three patients developed local recurrence while five patients developed distant recurrence (3 in the liver and 2 in the lung).

### Univariate and multivariate survival analysis

Tables 3 and 4 depicts the univariate and multivariate survival analysis of overall survival and progression-free survival. The univariate survival analysis in Table 3 illustrates that histological differentiation and T stage were associated with 3-year overall survival, while the multivariate survival analysis indicated that histological differentiation (HR = 6.978, 95% CI 1.534–31.795, *p* = 0.012) and T stage (*p* = 0.010) were independent prognostic factors of 3-year overall survival. Table 4 shows identical results where histological differentiation (HR 5.992, 95% CI 1.714–20.949, *p* = 0.005) and T stage (*p* = 0.004) were independent prognostic factors of 3-year progression-free survival.

**Table 3:** Univariate and multivariate survival analysis of overall survival.

Prognostic factor	Variates	No. (n = 94)	Univariate survival analysis			Multivariate survival analysis		
			HR	95% CI	*p*	HR	95% CI	*p*
Surgical approach	NOSE	49	0.799	0.215–2.977	0.738	1.252	0.298–5.336	0.750
	RARC	49						
Age (year)	≤ 60	68	1.735	0.466–6.463	0.411	1.620	0.419–6.355	0.492
	> 60	30						
Postoperative CEA (ng/ml)	≤ 5	47	0.835	0.649–1.075	0.162	0.335	0.078–1.404	0.135
	> 5	51						
Postoperative CA19-9 (U/ml)	≤ 30	67	0.252	0.032–2.017	0.194	0.252	0.031–2.179	0.211
	> 30	31						
T stage	I(ref)	9			**0.003**			**0.010**
	II	10	5.658	1.256–35.689		5.589	1.890–36.762	
	III	30	8.689	1.568–37.859		9.856	1.689–36.895	
Tumor size (cm)	≤ 3	24	1.116	0.232–5.377	0.891	1.356	0.257–7.165	0.720
	> 3	74						
Histological differentiation	Well + moderate	91	7.472	1.183–30.451	**0.005**	6.978	1.534–31.795	**0.012**
	Poor	7						

*p* < 0.05 values are indicated in bold.

*HR* hazard ratio, *95% CI* 95% confidence interval, *ref* reference.

**Table 4:** Univariate and multivariate survival analysis of progression-free survival.

Prognostic factor	Variate	No. (n = 94)	Univariate survival analysis			Multivariate survival analysis		
			HR	95% CI	*p*	HR	95% CI	*p*
Surgical approach	NOSE	49	1.009	0.379–2.688	0.986	1.525	0.535–4.412	0.421
	RARC	49						
Age	≤ 60	68	0.984	0.342–2.833	0.976	0.825	0.230–2.412	0.723
	> 60	30						
Postoperative CEA (ng/mL)	≤ 5	47	1.129	0.420–3.301	0.810	0.953	0.350–2.621	0.915
	> 5	51						
Postoperative CA19-9 (U/ml)	≤ 30	67	0.285	0.032–2.017	0.306	0.312	0.069–1.341	0.135
	> 30	31						
Tumor size (cm)	≤ 3	24	0.695	0.241–2.001	0.500	0.650	0.225–1.889	0.424
	> 3	74						
T stage	I (ref)	9			**0.002**			**0.004**
	II	10	4.158	1.125–30.289		5.023	1.680–35.026	
	III	30	6.589	1.368–32.589		9.856	1.465–36.568	
Histological differentiation	Well + moderate	91	5.902	1.836–18.979	**0.003**	5.992	1.714–20.949	**0.005**
	Poor	7						

*p* < 0.05 values are indicated in bold.

*HR* hazard ratio, *95% CI* 95% confidence interval.

## Discussion

This study is a case-matched based retrospective study. In this study, the surgical procedure of robotic rectal cancer resection with natural orifice extraction was introduced, after which the clinical outcomes were compared and univariate and multivariate survival analyses was performed. The present study recommends that robotic rectal cancer resection with natural orifice extraction is a safe and effective surgical modus operandi benefiting from better short-term outcomes, possessing considerable 3-year survival outcomes compared to conventional robotic assisted rectal cancer resection.

To our knowledge, this is the first study that compares the clinical outcomes and prognostic factors of robotic surgery. Previous studies have mostly focused on laparoscopic surgery with natural orifice extraction, and their conclusions mostly demonstrated that rectal cancer resection with natural orifice extraction benefited more from short-term outcomes. Efetov et al.^13^ reported that NOSE surgery possesses advantages in reducing the risk of intra-abdominal contamination and accurately identifying the line of rectal resection. In their case series, no intraoperative or postoperative complications occurred, and patient satisfaction was very high according to the CPGAS score results. Chen et al.^14^ suggested that NOSE surgery of the sigmoid colon and rectal tumors suffered less postoperative complications. Analogously, in terms of short-term outcomes, this study revealed the postoperative VAS scale on day 1 and postoperative use of analgesics were significantly less, and patients passed flatus quickly in the NOSE group. Many factors can affect the time to pass flatus; early activities out of bed can promote the recovery of gastrointestinal function. In the NOSE group, patients suffered less abdominal pain and could get out of bed earlier, hence, postoperative use of analgesics and time to pass flatus were less. The distance from the tumor margin to the anus was observed in the NOSE group, which was on a significantly longer margin than that in the RARC group. In the NOSE group, the rectum was first transected by ultrasound knife in order to extract the sterile plastic sheath, after which the distal rectal opening was closed using a linear stapler, which may have caused the distance from the tumor margin to the anus to be longer than that in the RARC group. Although it is not strictly statistically significant, this may have caused the relatively small size.

The stress response to surgery results in inflammation, which varies in accordance to the level of injury^15,16^. Theoretically, the more severe the inflammation, the more difficult it is for the wound and body to recover^17^. Besides, an increasing amount of evidence suggests that the stress response caused by surgery may promote the growth of pre-existing micro-metastasis or potentially initiate tumors^18,19^. No current agreed upon indicators exist in measuring the inflammation level. However, white blood cell count, C-reactive protein and procalcitonin remain the mostly used indexes^20,21^. This study showed that white blood cell count and postoperative procalcitonin increased in both groups on postoperative days 1 and 3, dropping to normal levels on postoperative day 5. However, postoperative C-reactive protein in the NOSE group was at significantly lower levels on postoperative days 1 and 3, similar to Zhou’s study^6^. The reduced degree of abdominal incisions may account for this result, demonstrating that transanal natural orifice specimen extraction surgery reduced surgical stress response to some extent. Moreover, patients in the NOSE group had significantly less postoperative VAS scale on day 1, which may have been due to the reduced surgical stress response. Several reasons may account for patients in the NOSE group to pass flatus quickly. The two most important reasons were that patients in NOSE group did not have abdominal incisions and released less inflammatory mediators to disrupt gastrointestinal function.

Histological differentiation and T stage were calculated as independent prognostic factors for 3-year overall survival and 3-year progression-free survival. Patients with well and moderate differentiation had a significantly lower survival than patients with poor differentiation. The higher the T stage, the worse the prognosis. However, this study was confined to a small population, hence, in order to acquire a more convincing and stratified conclusion regarding the prognostic factors of robotic rectal cancer resection, a larger population study is required.

This study has some limitations. To reduce selection bias, matching cases was done according to confounding factors. This study collected data at a single center, and the surgeon’s background may affect the results to some extent. Rectal cancer resection with natural orifice extraction surgery is limited to tumor size, and bulky tumors are not appropriate for surgery in this manner. Moreover, this study is limited by its small size. To better understand the safety and feasibility of robotic rectal cancer resection with natural orifice extraction, a large-scale population randomized study is required. To this effect, we have registered a multicenter, randomized clinical trial (NCT04230772) and are currently recruiting participants.

## Conclusion

Robotic rectal cancer resection with natural orifice extraction is a safe and feasible minimally invasive surgery used to treat patients with rectal cancer as it possesses considerable advantages such as decreased pain, quicker recovery of intestinal function, and reduced surgical stress response. Furthermore, the long-term survival analysis showed no differences between the two groups, and histological differentiation and T stage could be regarded as independent prognostic factors for 3-year overall survival and 3-year progression-free survival.
